# Health-related quality of life in breast cancer patients: review of reviews from 2008 to 2018

**DOI:** 10.1186/s12955-020-01591-x

**Published:** 2020-10-12

**Authors:** Parisa Mokhtari-Hessari, Ali Montazeri

**Affiliations:** 1 Integrative Oncology Research Group, Breast Cancer Research Center, Motamed Cancer Institute, ACECR, Tehran, Iran; 2 Population Health Research Group, Health Metrics Research Center, Iranian Institute for Health Sciences Research, ACECR, Tehran, Iran; 3 Faculty of Humanity Sciences, University of Science and Culture, ACECR, Tehran, Iran

**Keywords:** Overview, Breast cancer, Quality of life, Review of reviews, Patient-reported outcome

## Abstract

**Background:**

Breast cancer still is a topic. This overview of the literature aimed to update the current knowledge on quality of life in breast cancer patients.

**Methods:**

A review of literature in MEDLINE, Cochrane Database of Systematic Reviews and Google Scholar were carried out to identify review papers on health-related quality of life in breast cancer during the 2008 to 2018. All publications were screened using the PRISMA guideline. The methodological quality of reviews was assessed using the AMSTAR. The findings were summarized and tabulated accordingly.

**Results:**

Within over a decade, a total of 974 review papers were identified which according to the study selection criteria finally we have evaluated 82 reviews. Of these about 85% had a reasonable methodological quality. The findings were mainly summarized on several headings including instruments used to measure quality of life, treatment, supportive care, psychological distress, and symptoms. Questionnaires had a good performance to quantify quality of life in breast cancer patients. Most reviews were focused on the impact of treatment including endocrine therapy as well as integrating complementary and alternative medicine into the current practice. According to the reviews, yoga was the most recommended exercise to improve quality of life in breast cancer patients.

**Conclusion:**

Overall, the findings from this overview indicated that quality of life in breast cancer patients enhanced during the last decade. Several simple but effective interventions such as physical activity and psychosocial interventions proved to be effective in improving quality of life in this population. However, management of symptoms such as pain, and lymphedema, issues related to worry, sexual function especially for young patients, and the future outlooks all are among topics that deserve further consideration. Also, this overview indicated that methodological issues in measuring quality of life in breast cancer patients improved greatly, but still there is a long way to go to understand what really matter to patients.

## Background

Breast cancer remains the most common cancer among women worldwide [1]. According to 2018 GLOBOCAN, approximately 2.1 million cases worldwide were diagnosed with breast cancer and about 630,000 died from the disease [2]. Due to the increasing in breast cancer incidence, advances in the treatment of the disease have been achieved. Local modalities and systemic anticancer therapies, therefore, lead to improve patients’ survival outcomes including disease-free survival and overall survival [3]. However, since the disease diagnosis and treatment have improved greatly over time, at present in addition to survival, quality of life has become an important outcome measure in breast cancer clinical investigations and survivorship studies [4, 5]. Hopefully, at present a compile of evidence exist on the topic and sometimes even it is very difficult to adhere to evidence in practice since conflicting findings are reported. Thus, to evaluate and summarize the existing evidence on quality of life in breast cancer patients a review of reviews was conducted.

Previously we have summarized all reviews on breast cancer patients’ quality of life that covered the literature from publication of the first review up to year 2008 [6]. The lists of those reviews are supplemented (see Additional file 1). Now we are updating the review by focusing on review papers which appeared in biomedical journals since then. Systematic review of reviews will allow the creation of a summary of reviews in a single document in order to enhance evidence-based knowledge and support well-informed clinical decision-making [7]. The present review of reviews aimed to address the primary question of whether the quality of life has been improved over the last decade and what factors have played the key role in patients’ quality of life. In fact, the goal of this review of reviews was to identify the impact of breast cancer and its treatment on quality of life and to determine ways to improve quality of life in breast cancer patients.

## Methods

### Definition

Quality of life or specifically health-related quality of life was defined as breast cancer patients’ perception of their own physical, mental and social health that influenced by diagnosis, treatment, post-treatment, and survivorship as assessed by using well validated instruments.

### Search engines and time period

Studies identified through the available literature in MEDLINE (PubMed), and Goggle scholar to identify review papers on health-related quality of life in breast cancer. Also, an extra search was performed to check reviews indexed in the Cochrane Database of Systematic Reviews (CDSR). Current study covers all full review publications that appeared in English language biomedical journals between January 2008 and 31 December 2018.

### Search strategy

This study used comprehensive evidence map of overview of systematic reviews introduced by Lunny et al. [8]. All databases were searched using the combination of keywords ‘quality of life’ and ‘breast cancer’ or ‘breast carcinoma’ in the titles of publications and limited to review articles. This provided the initial database for the review. Initial search was carried out in late January 2017, twice on March, and August 2017 and once for a final update on February 2019. A manual search also was performed for possible additional references. Key words and search strategy were as follows: (breast cancer [Title/Abstract]] AND quality of life [Title/Abstract] Filters: Review; Publication date from 2008/01/01 to 2018/12/31; English).

### Selection criteria

Eligibility criteria for inclusion were: all review papers that published in English language, and reviewed quality of life as a main outcome in breast cancer patients. All other papers were excluded. All publications were screened using the PRISMA guideline. The AMSTAR checklist is used to assess the quality of reviews [9].

### Data synthesis

Data obtained from each single review were synthesized by providing descriptive tables reporting authors’ names, publication year, type of review, number of databases and studies included, analysis, and whether performed quality appraisal and risk of bias assessments. The findings were presented chronologically.

## Results

### Statistics

**Fig. 1:**
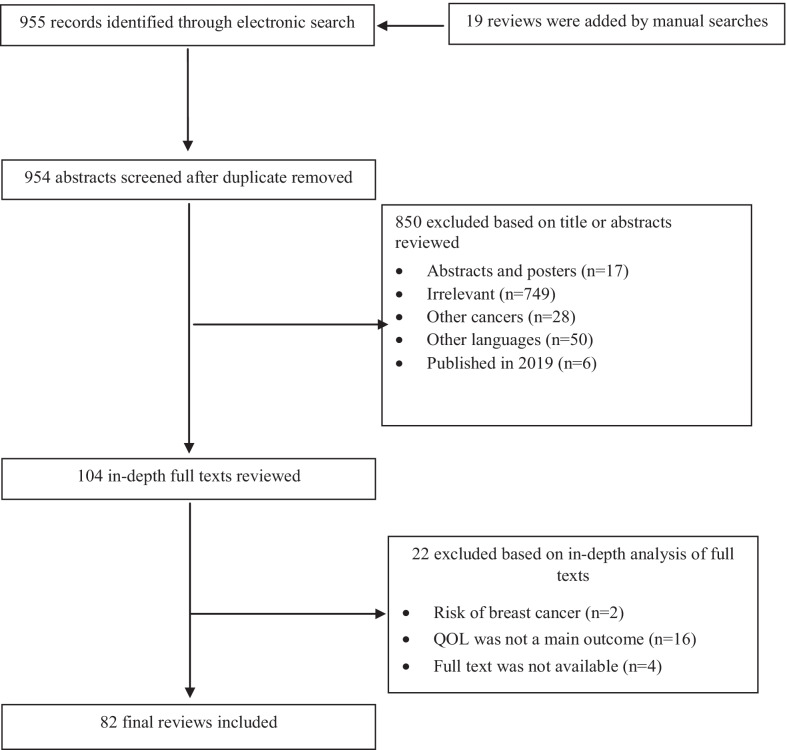
The study flowchart

**Fig. 2:**
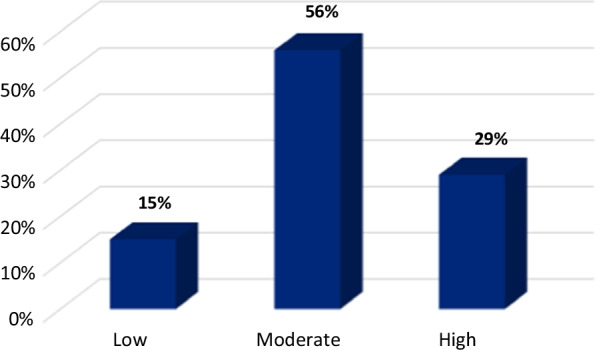
Quality scoring according to the AMSTAR checklist

A total of 955 unique review articles were identified. In addition, 19 citations were found via manual search (n = 974). After removing duplicates, commentaries and brief communications, 104 reviews seemed relevant for further evaluation. Finally, of these 81 quantitative and one qualitative review were found eligible and included in the study. The study flowchart is shown in Fig. 1. According to the AMSTAR checklist, approximately 85% of the publications had value of 4 or more for methodological quality (Fig. 2).

### Overall outlook of reviews

**Fig. 3:**
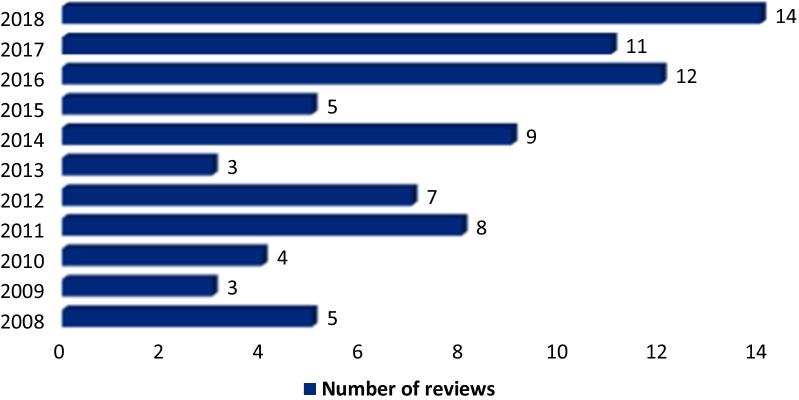
Frequency of reviews on quality of life in breast cancer patients during 2008–2018

In general, although not having the same quality, currently reviews, systematic reviews and meta-analyses regarding QOL in breast cancer patients are increasing (Fig. 3). However, to summarize evidence, reviews were categorized into the following main topics: reviews on measurements and methodological issue, reviews that dealt with different treatments, and those reviews that touched other topics such as supportive care, physiological distress, age-related reviews, quality of life in different nations/races and qualitative reviews. These are presented in the following sections.

#### Quality of life measurement

1. Instruments used

There were 17 papers that reviewed literature on instruments to quantify quality of life in breast cancer patients [10–26]. In general, there were three types of instruments: generic, specific, and measures assessing psychological issues or breast cancer related symptoms. Among generic measures the Short Form Health survey (SF-36) and the brief version of World Health Organization Quality of Life Questionnaire (WHOQOL-BREF) had a good performance [20]. Also, the European Organization for Research and Treatment of Cancer quality of life core cancer (EORTC QOL-C30) questionnaire and the Functional Assessment of Cancer Therapy/Functional Assessment of Chronic Illness Therapy (FACIT) were the most commonly used questionnaires [12, 13]. Reviews also found that specific measures including the Functional Assessment of Cancer Therapy-Breast quality-of-life (FACT-B) and the European Organization for Research and Treatment of Cancer quality of life core breast cancer (EORTC QLQ-BR23) were the frequently used specific QOL instruments in breast cancer patients [13, 18–20]. The FACT-ES and the Hot Flash Related Daily Interference Scale (HFRDIS) had good applicability for patients who receive hormonal treatment and who have hot flashes [17]. A systematic review of QOL instruments in long-term BCS indicated that the Quality of Life in Adult Cancer Survivors Scale has acceptable reliability, validity, and responsiveness [14]. The findings are summarized in Table 1.

(2) The challenges exist

**Table 1:** A list of reviews on measurement issue and quality of life in breast cancer patients (2008–2018)

Authors [References]	Year	Main focus	Description/analysis	No. of databases	No. of included studies	Performed QA	Risk of bias assessment	Result(s)
Chen et al. [10]	2010	Patient-reported outcome measures for oncologic breast surgery	Systematic review	8	NA	No	No	Reliable and valid instruments exist, but even the best instruments do not address all important surgery‐specific and psychometric issues
Winters et al. [11]	2010	Treatment recommendations in breast reconstruction based on patient-reported outcome measures and HRQOL	Systematic review	4	34	Yes	No	Sound scientific methodology in HRQOL is undermined by poorly designed and underpowered studies There is a pressing need for further longitudinal studies in breast reconstruction incorporating sensitive and condition-specific patient-report outcome measures; provide adequate sample sizes, and respect established guidelines for rigorous HRQOL methodology
Lemieux et al. [12]	2011	QOL measurement in RCTs	An updated systematic review	PubMed	190	No	No	Reporting of QOL methodology should improve
Reed et al. [13]	2012	QOL assessments in advanced breast cancer	Review	PubMed	51	No	No	There should be more consensuses on which QOL instruments are used
Chopra and Kamal [14]	2012	QOL instruments in long-term BCS	Systematic review	5	19	No	No	The use of validated instruments will not only provide valid data but also help improve the quality of care in long-term BCS
Adamowicz et al. [15]	2012	Assessment of HRQOL parameters as end points in phase III trials in advanced BC	Review	NA	34	No	No	HRQOL evaluation in clinical trials has the potential to predict patient prognosis and serves as a useful tool to assess patients’ experience during cancer therapy
Pusic et al. [16]	2013	Patient-reported outcome instruments and outcomes in breast cancer patients with lymphedema	Systematic review	NA	39	Yes	No	The Upper Limb Lymphedema 27 (ULL-27) was found to have strong psychometric properties. Future studies should strive to use high-quality condition- specific PRO instruments, follow existing guidelines for HRQOL measurement
Niu HY et al. [17]	2014	Validity, reliability and responsiveness of breast cancer-specific HRQL instruments	Review	3	4	Yes	No	The EORTC QLQ-BR23, FACT-B, FACT-ES, and HFRDIS had fairly good psychometric properties to assess HRQOL
Nguyen et al. [18]	2015	Effectiveness of EORTC QLQ-BR23 and FACT-B	Review	3	NA	No	No	Both questionnaires were effective in assessing QOL. Decision-making between the questionnaires depends on the study’s purpose and design
Oliveira et al. [19]	2015	The procedures of translation, cross-cultural adaptation, and measurement properties of breast cancer-specific QOL questionnaires	Systematic review	4	24	Yes	No	Caution should be exercised when using breast cancer-specific QOL questionnaires that have been translated, adapted, and tested
Maratia et al. [20]	2016	Evaluation of available specific and generic breast cancer HRQOL instruments	Systematic review	2	32	Yes	No	The EORTC BR-23, IBCSG, SF-36, and WHO-QOL BREF had good performance, depending on the purpose of the study
Ghislain et al. [21]	2016	HRQOL in locally advanced and metastatic breast cancer: reporting of methodological and clinical issues in RCTs	Systematic review	PubMed	49	Yes	No	The absence of the HRQOL research hypotheses and the overemphasis on statistical rather than clinical significance was the main problem
Turner-Bowker et al. [22]	2016	Patient-reported outcomes in advanced breast cancer clinical trials	Systematic review	3	25	No	No	Patient-reported outcomes may be used to provide a more comprehensive perspective of the benefits and risks from treatment
Krohe et a l [23]	2016	PRO in metastatic breast cancer: a review of industry-sponsored clinical trials	Review	Clinicaltrial.gov	38	No	No	Stakeholders turn more attention to the patient perspective; one would expect PROs to increase as complementary measures to traditional endpoints and become an even more critical part of treatment evaluation
Pe et al. [24]	2018	Patient-reported outcome data in randomized controlled trials of locally advanced and metastatic breast cancer	Review	PubMed	66	No	No	A need to improve standards in the analysis, interpretation, and reporting of Patient-reported outcome and quality of life data in cancer RCTs
Liu et al. [25]	2018	BREAST-Q measurement of the patient perspective in oncoplastic breast surgery	Systematic review	4	54	Yes	No	BREAST-Q can effectively measure patient's satisfaction and HRQOL in relation to different type of breast oncoplastic surgeries. BREAST-Q captured meaningful and reliable information from the patients' perspective and may be useful for clinical decision-making
Tevis et al. [26]	2018	Patient-reported outcomes for breast cancer	Review	Not specified	123	No	No	The implementation of PROs can be complex and challenging and care must be taken to minimize the potential for survey fatigue by patients and the potential financial burden for implementation, maintenance, and analyses of collected data

*QA* quality appraisal, *NA* not available, *QOL* quality of life, *HRQO* health-related quality of life, *PRO* patient-reported outcomes, *RCTs* randomized clinical trials, *EORTC QLQ-C30* the European Organization for Research and Treatment of Cancer Quality of Life Questionnaire-Core, *FACT-B* The Functional Assessment of Cancer Therapy-Breast quality-of-life, *EORTC QLQ-BR23* the European Organization for Research and Treatment of Cancer quality of life core breast cancer, *HFRDIS* Hot Flash Related Daily Interference Scale, *IBCSG* the International Breast Cancer Study Group, *SF-36* The 36-Item Short Form Health Survey, *WHO-QOL BREF* World Health Organization Quality of Life Instruments, *FACT-ES* Functional Assessment of Cancer Therapy-Endocrine Subscale, *BREAST-Q* patient-reported outcome measure for breast surgery

Three papers critically reviewed the literature and pointed out that some shortcomings exist among studies reporting quality of life in breast cancer patients. As such a review on quality of life in breast cancer patients who received breast conservation surgery echoed that instruments do not address all important surgery‐specific and psychometric issues of oncologic breast surgery patients [10]. Similarly, a systematic review conducted to guide treatment recommendations in breast reconstruction based on patient-reported outcomes and HRQOL revealed that sound scientific methodology in HRQOL were undermined by poorly designed and underpowered studies. The review recommends that studies on the topic ‘should incorporate sensitive and condition-specific patient-report outcome measures, provide adequate sample sizes, and respect established guidelines for rigorous HRQOL methodology’ [11].

Recently a review including 49 RCTs in locally advanced and metastatic setting concluded that the absence of QOL research hypotheses and overemphasis on statistical than clinical significance is still problematic in measuring quality of life in breast cancer patients. The authors pointed out that ‘although most of the experts’ recommendations have been broadly followed by the research community during the past decade, the specification of the HRQOL research hypothesis is still under-reported’ [21].

#### Treatment

A summary of reviews that focused on different treatment modalities and quality of life are presented in Table 2 [27–54].

1. Systemic therapy

**Table 2:** A list of reviews covering local, systemic treatments and side-effects and quality of life in breast cancer patients (2008–2018)

Authors [References]	Year	Main focus	Description/analysis	No. of databases	No. of included studies	Performed QA	Risk of bias assessment	Result(s)
Lemieux et al. [27]	2008	Effects of Chemotherapy-induced alopecia on QOL	Review	5	38	No	No	Hair loss consistently ranked amongst the most troublesome side effects, was described as distressing, and may affect the body image
Cella and Fallowfiel [28]	2008	Side-effects of endocrine therapy and QOL	Review	NA	6	No	No	Starting with better QOL, which should lead to better adherence, will result in better patient outcomes
Buijs et al. [29]	2008	Endocrine treatments for breast cancer and HRQOL	Review	NA	NA	No	No	HRQOL mostly is severely influenced by chemotherapy and part of these symptoms may be lasting, especially when associated with the induction of premature menopause. The varying side effect profiles of tamoxifen and aromatase inhibitors did not lead to significant difference in overall HRQOL
Pockaj et al. [30]	2009	QOL after breast surgery	Review	NA	NA	No	No	Better preoperative counseling, informed decision-making, and appropriate interventions will lead to improved QOL
Reimer and Gerber [31]	2010	Impact of local or systemic treatments on QOL in the elderly early-breast cancer patients	Review	PubMed	18	No	No	Overall QOL was maintained or improved
Devi et al. [32]	2011	QOL of women during and up to ten years after treatment for breast cancer	Systematic review	9	11 qualitative studies	Yes	No	Breast cancer diagnosis and its treatment can have a significant effect on several domains of women’s QOL
Pinto and de Azambuja [33]	2011	Symptoms in BCS	Review	NA	NA	No	No	The most common symptoms affecting BCS were fatigue, insomnia, depression, cognitive dysfunction, reproductive and menopausal symptoms and lymphedema
Howard-Anderson et al. [34]	2012	QOL, menopausal symptoms and fertility concerns, and behavioral health outcomes in younger patients	Systematic review	PubMed	28	No	No	Younger women experienced concerns on premature menopause, menopausal symptoms and infertility that had a role in the level of distress after treatment. Health outcomes in younger ones include weight gain and physical inactivity
Kaviani et al. [35]	2013	Type of surgery and QOL	Narrative review	NA	NA	No	No	QOL had a better score for oncoplastic breast surgery in comparison with mastectomy or BCT
Orsaria et al. [36]	2014	Nodal status assessment and QOL	Review	NA	NA	No	No	Quality results in breast cancer surgery need to generate oncological safety devoid of complications through renewed clinical experience
Tsoi et al. [37]	2014	Tissue-expander/implant vs. autologous abdominal tissue breast reconstruction	Systematic review	5	15	Yes	Yes	There is some weak evidence that tissue-expander/implant reconstruction becomes a less favorable approach in terms of patient satisfaction after mastectomy
Taghian et al. [38]	2014	Impact of lymphedema on QOL	Review	PubMed	NA	No	No	Lymphedema remains a significant QOL issue which affected woman’s physical, psychological, and emotional well-being
Sodergren et al. [39]	2015	The side effects associated with targeted therapies used in the adjuvant and metastatic settings for HER2+	Systematic review	5	18	No	No	Compared with conventional cytotoxics, targeted therapies are delivered over longer periods of time and present unique and often extensive side-effect profiles. Diarrhoea and skin rash as particularly prevalent anti-HER2 inhibitor side effects
Kameo and Sawada [40]	2016	QOL and adverse reactions of chemotherapy	Integrative review	5	50	NA	NA	Multidisciplinary and thinking on the individual vulnerabilities should be considered when evaluating adverse reaction and QOL
Mioranza et al. [41]	2016	The impact of adjuvant endocrine therapy in early breast cancer on QOL	Review	NA	NA	No	No	Impact of adjuvant endocrine therapies on HRQOL was not comparable since they used different QOL instruments
Razdan et al. [42]	2016	Quality of life among patients after bilateral prophylactic mastectomy	Systematic review	6	22	Yes	No	Post-BPM, patients are satisfied with the outcomes and report high psychosocial well-being and positive body image. Sexual well-being and somatosensory function are most negatively affected. Vulnerability, psychological distress and preoperative cancer distress are significant negative predictors of quality of life and body image post-BPM
Chalasani [43]	2017	Optimizing QOL in patients with hormone receptor-positive metastatic breast cancer	Review	NA	NA	No	No	Patients with HR-positive disease may receive maximum clinical benefit from endocrine therapy while optimizing QOL
Garrido-Oyarzun and Castelo-Branco [44]	2017	Use of hormone therapy for menopausal symptoms and QOL in survivors	Review	NA	NA	No	No	The management of menopausal symptoms and QOL of patients treated for breast cancer remains an important problem without an optimal solution
Marta et al. [45]	2017	QOL in patients treated with radiation therapy	Systematic review	PubMed	353	No	No	Significant benefit in HRQOL was often reported when a positive primary outcome was reported
Zhou et al. [46]	2017	Impact of endocrine monotherapy and in combination with targeted therapy on QOL	Systematic review	3 databases and key conferences	11	No	No	Users of both treatments experienced similar QOL in the first-line and ET-failure setting relative to patients on ET mono. Moreover, these users experienced better QOL outcomes in some domains in the ET-failure setting relative to ET mono users
Mileski et al. [47]	2017	QOL considerations during cancer treatment in invasive ductal carcinoma patients	Systematic review	PubMed	9	No	No	The most prevalent positive QOL factors included patient expectations, decreased side effects, and increased survival rate. The most prevalent negative QOL factors included treatment, specific side effects and decreased quality of life
Platt and Zhong [48]	2017	Patient-centered breast reconstruction based on health-related QOL evidence	Review	NA	NA	No	No	Both immediate and delayed breast reconstruction increased satisfaction and QOL after reconstruction, and both groups have reported similar satisfaction and QOL scores at long-term follow-up
Rivera et al. [49]	2018	Chemotherapy-associated peripheral neuropathy	Systematic review	4	60	Yes	No	Neuropathic symptoms persisted in 11.0% to more than 80% of participants at one to three years following treatment. There is a paucity of data describing persistent PN in ESBC patients
Xiao et al. [50]	2018	Effects of adjuvant endocrine therapy on the QOL of post-menopausal women with non-metastatic ER+	Systematic review	3	13	Yes	No	Most studies found no differences between tamoxifen and aromatase inhibitor groups in terms of global QOL. The QOL of post-menopausal women is unlikely to be adversely affected by long-term use of adjuvant endocrine therapy
Yee et al. [51]	2018	Radiation-induced skin toxicity in breast cancer patients	Systematic review	NA	96	Yes	No	Methods including simultaneous integrated boost, accelerated partial breast irradiation, and prone positioning may cause less radiation dermatitis than conventional treatments
Cheng et al. [52]	2018	QOL of elderly patients with solid tumors undergoing adjuvant cancer therapy	Systematic review	5	4	Yes	Yes	Adjuvant chemotherapy and radiotherapy may not have detrimental effects on QOL in most elderly patients with solid tumors
Muller et al. [53]	2018	Impact of manual lymphatic drainage on the HRQOL	Systematic review	6	8	Yes	Yes	No studies reported reductions in HRQOL, or severe adverse events after MLD
Jeffs et al. [54]	2018	Effectiveness of decongestive treatments on excess arm volume and patient-centered outcomes in women with early breast cancer-related arm lymphedema	Systematic review	3	7	Yes	Yes	Weak evidence for the impact of decongestive lymphedema treatment did not allow any conclusions to be drawn about the most effective treatment to be offered when patients with early breast cancer first present for treatment

*QA* quality appraisal, *NA* not available, *QOL* quality of life, *HRQO* health-related quality of life, *BPM* body image post, *ET* endocrine therapy, *BCT* breast conserving therapy, *HER_2* human epidermal growth factor receptor 2, *RCTs* randomized clinical trials, *BCS* breast cancer survivors, *MLD* impact of manual lymphatic drainage

Seventeen reviews were focused on HRQOL in patients undergoing systemic therapy including chemotherapy, hormonal therapy, and targeted therapy. Of these, the effect of endocrine therapy alone or combined with adjuvant treatments was the center of focus. Hot flashes were the most common side effect of adjuvant endocrine therapies. Side effects of tamoxifen and aromatase inhibitors including vaginal dryness, vaginal discharge, dyspareunia, and arthralgia were often reported in reviews [28]. A review assessing the impact of adjuvant endocrine therapy in early breast cancer on QOL found that in most trials, treatment-related symptoms led to the small drop in different domains of QOL [41].

Despite the current guidelines considering that hormonal therapy is contraindicated in breast cancer survivors, recently a review suggested that in some women, menopausal symptom relief might be more important than the potential risks of hormonal therapy. The review concluded that on the use of hormonal therapy and tibolone in newly diagnosed patients, survivors or suspected to breast cancer will remain contraindicated [44].

(2) Local therapy including surgery and radiotherapy

Six reviews addressed the impact of local therapies on quality of life [30, 35–37, 42, 48]. For example, one review found that there was worse body image, disturbances in sexual life as well as great impairment in advanced breast cancer patients after mastectomy [42]. Patients receiving immediate and delayed breast reconstruction experienced increased satisfaction and QOL after reconstruction, and in long-term follow-up, both groups have reported similar satisfaction and QOL scores [48]. Marta et al. found that HRQOL has been infrequently investigated in RCTs in patients who received radiotherapy. QOL can be an important predictor of better treatment outcomes, as significant benefit in HRQOL was often reported once a positive primary outcome was reported [45].

(3) Side effects of treatments

Five reviews focused on side effects of treatment modalities including topics related to fatigue, insomnia, cognitive dysfunction, reproductive and menopausal symptoms and lymphedema [33, 34, 38, 53, 54]. A review of randomized clinical trials found that these symptoms were the most common symptoms affecting survivors’ quality of life [33]. Lymphedema in early-stage breast cancer patients who undergo axillary lymph-node dissection is an important concern. The results derived from a total of 8 studies have shown that impact of manual lymphatic drainage had a significant impact on HRQOL, but a recent published review failed to find that the impact of decongestive lymphedema treatment, the most effective treatment to be offered, on patients with early lymphedema due to the weak evidence [53, 54].

#### Supportive care

In the following sections we highlighted a number of topics relevant to supportive care in breast cancer patients [55–72]. The findings are summarized in Table 3.

1. Physical activity (supportive exercise intervention)

**Table 3:** A list of reviews on supportive care including physical activity, complementary and alternative medicine and quality of life (2008–2018)

Authors [References]	Year	Main focus	Description/analysis	No. of databases	No. of included studies	Performed QA	Risk of bias assessment	Result(s)
Bicego et al. [55]	2009	The effect of exercise on QOL	Systematic review	4	9	Yes	No	Exercise as an effective strategy can improve QOL in patients
Bleakley and Stinson [56]	2011	CAM and QOL	Narrative review	6	8	Yes	No	There is great potential for complementary and alternative therapies to be increasingly integrated into clinical practice within breast cancer services
Levine and Balk [57]	2012	Yoga and QOL improvement	Literature review	7	71	NA	NA	Participation in yoga programs appeared to benefit breast cancer patients
Zhang, et al. [58]	2012	Effects of yoga on psychological function and QOL	Systematic review and meta-analysis	5	6	Yes	Yes	There is insufficient evidence to advocate that yoga should be offered routinely to women diagnosed with breast cancer. However, it may be an acceptable intervention to improve QOL for these women
Boehm et al. [59]	2014	Arts therapies for anxiety, depression, and QOL	Systematic review and meta-analysis	3	13	Yes	Yes	Overall, the option of participation in arts therapies can be recommended and has shown to be significantly effective for the reduction of anxiety in patients
Sawyer [60]	2014	Complementary exercise and QOL	Systematic review	4	9	No	No	Although complementary exercise improved QOL statistically in two-thirds of the research findings, further research is recommended
Zeng Y et al. [61]	2014	Effects of exercise intervention on QOL in BCS	Systematic review and meta-analysis	5	19	Yes	Yes	Exercise interventions have statistically significant effects on overall QOL, as well as positive trends for breast and arm symptoms
Yan et al. [62]	2014	Lack of Efficacy of Tai Chi in Improving QOL in BCS	Systematic review and meta-analysis	4	9	Yes	Yes	There is a lack of sufficient evidence to support Tai Chi benefiting the management of BCS in improving QOL and other important clinical outcomes
Leggett et al. [63]	2015	Effects of CAM on cancer symptoms, treatment side effects, QOL, and survival	Systematic review	5	22	Yes	Yes	Guarana and Ganoderma lucidum may improve fatigue, whereas glutamine may also be effective in improving oral mucositis symptoms
Van Dijck et al. [64]	2016	The effects of different physical self-management techniques on QOL	Systematic review	4	13	Yes	Yes	Physical self-management interventions during breast cancer treatment as well as after the primary treatment seem to generate beneficial effects on QOL
Zhang et al. [65]	2016	Effects of mindfulness-based therapy on QOL	Systematic review and meta-analysis	6	7	Yes	Yes	There was limited that mindfulness-based therapy can improve QOL
Cramer et al. [66]	2017	Yoga for improving HRQOL, mental health and cancer-related symptoms	Systematic review and meta-analysis	6	23	Yes	Yes	Moderate-quality evidence supported the recommendation of yoga for improving HRQOL and reducing fatigue and sleep disturbances when compared with no therapy, as well as for reducing depression, anxiety and fatigue, when compared with psychosocial/educational interventions
D'Egidio et al. [67]	2017	Effect of counseling interventions on HRQOL	Systematic review	2	35	Yes	No	Exercise counseling as well as physical therapy are effective to improve shoulder mobility, healing wounds, and limb strength
Husebo et al. [68]	2017	Mind–body exercise therapies and QOL	Review	4	11	Yes	Yes	Yoga was found to benefit patients’ psychological QOL, while less support was established concerning physical QOL elements
Lipsett et al. [69]	2017	Exercise during adjuvant radiotherapy on fatigue and QOL	Systematic review and meta-analysis	6	9	Yes	No	Statistically significant benefits of supervised, combined aerobic resistance exercise on fatigue were achieved
Pan et al. [70]	2017	Yoga practice improve treatment-related side effects and QOL	Systematic review and meta-analysis	3	16	Yes	No	Yoga was associated with enhanced overall HRQOL and relief of anxiety, depression and gastrointestinal adverse reactions in breast cancer patients and survivors
Zaidi et al. [71]	2018	Effect of complementary therapies on survivors’ QOL	Review	NA	NA	No	No	There is a need for personalized physical activity plans to be developed to suit the individual and their circumstances
Zhang et al. [72]	2018	Effectiveness of telephone- based interventions on HRQOL and prognostic outcomes	Systematic review and meta-analysis	6	14	Yes	Yes	Based on the insufficient evidence, the effects on depression, fatigue and physiological function were not statistically significant

*QA* quality appraisal, *NA* not available, *CAM* complementary and alternative medicine, *QOL* patient-reported outcomes, *BCS* breast cancer survivors, *HRQOL* health-related quality of life

There were 6 systematic reviews on physical activity and quality of life in breast cancer patients. Overall, evidence suggests that physical activity could enhance quality of life and reduce symptoms [55, 58, 60, 61, 64, 69]. For instance, a meta-analysis consisting of 5544 patients found that exercise interventions such as aerobic, Tai Chi, yoga, stretch training, and resistance training in survivors had statistically significant effects on overall QOL and breast and arm symptoms [61].

(2) Complementary and alternative medicine (CAM)

A variety of reviews assessed the effect of complementary and alternative medicine including diet and dietary supplements, energy therapies, manipulative and body-based practices, and mind–body techniques on the QOL aspects. Reviews on the effect of CAM on symptoms showed a significant improvement in symptoms [63]. One study of reviewing publications targeted mind–body exercise including yoga, Tai Chi chuan, and qigong found that breast cancer patients’ psychological quality of life benefited from yoga, while physical elements of quality of life were not supported [68]. Yoga is the most studied mind–body therapy. Reviews focusing on the effect of yoga on quality of life among survivors showed that although the literature provided evidence of health related quality of life benefits or significant effects of yoga on reducing fatigue and sleep disturbances, for example, [58, 66, 70], any specific aspect of yoga was not recognized as being most advantageous [57]. The results of a meta-analysis including 951 patients on mindfulness-based therapy on QOL aspects indicated an improvement of this therapy on reducing anxiety, depression, fear of recurrence, and fatigue associated with breast cancer [65]. However, a systematic review of the effect of art therapies on anxiety and depression indicated that such interventions could have a significant effect on patients’ reduced anxiety [57].

#### Psychological distress

Reviews concerning psychological issues and quality of life are presented in Table 4 [73–79]. Psychoeducational support found to be effective in improving breast cancer symptoms and emotional well-being among breast cancer patients [76]. In addition, a review found that reported psychological distress including anxiety and depression were common among breast cancer patients [75] and the treatment of depression could have an important role on improving QOL and increasing longevity [74].

**Table 4:** A list of  reviews on psychological distress and quality of life (2008–2018)

Authors [References]	Year	Main focus	Description/analysis	No. of databases	No. of included studies	Performed QA	Risk of bias assessment	Result(s)
Reich et al. [73]	2008	Impact of depression on QOL	Review	NA	NA	No	No	Treatment of depression improves QOL and may increase longevity
Duijts et al. [74]	2011	Effect of behavioral techniques and physical exercise on psychosocial functioning and HRQOL outcomes	Systematic review and meta-analysis	6	56	No	No	Behavioral techniques and physical exercise improve psychosocial functioning and HRQOL in breast cancer patients and survivors
Paraskevi [75]	2012	QOL outcomes	Review	4	NA	No	No	Psychological distress-anxiety and depression were common among BC patients. Pain, fatigue, and insomnia were the most common symptoms reported
Matsuda et al. [76]	2014	Effectiveness of psychoeducational support on quality of life	Systematic review and meta-analysis	CENTRAL	8	No	Yes	Psychosocial support in improving BC symptoms and in improving emotional well-being is effective within 6 months post-intervention
Chow et al. [77]	2016	Body Image and QOL	Review	5	13	NA	NA	BC survivors were reported a poorer body image and deterioration in their QOL after treatment. There was not enough evidence of the correlation between body image and QOL
Ye et al. [78]	2018	Efficacy of cognitive behavior therapy on QOL and psychological health of survivors	Meta-analysis	4	10	Yes	Yes	Due to the effectiveness of therapy for psychological symptoms, cognitive behavior therapy should be used as the intervention
Abrahams et al. [79]	2018	Relationship of fatigue with QOL and factors that can be addressed in psychological interventions	Systematic review	5	57	Yes	No	Moderate to strong evidence appeared for a relationship of fatigue with depressive symptoms, anxiety, distress, sleep disturbances, lower physical activity levels, pain, difficulties with coping with cancer, and catastrophizing about symptoms

*QA* quality appraisal, *NA* not available, *QOL* quality of life, *HRQOL* health-related quality of life, *NA* not applicable, *CENTRAL* Cochrane Central Register of Controlled Trials, *BCS* breast cancer survivors

#### Age-related reviews

Descriptive characteristics of reviews concerning quality of life in young and elderly breast cancer patients are summarized in Table 5 [80–83]. A review on long-term survivors indicated that it seems older patients are better prepared mentally to deal with treatments, despite of having co-morbidities [80]. While a study on young survivors reported greater fear of death, unmet supportive care needs, financial constrain, and minor physical well-being. Spiritual well-being aspects seemed favorable among this subpopulation. However, these patients generally experience suboptimal HRQOL after breast cancer diagnosis [83].

**Table 5:** A list of age-related reviews on quality of life in breast cancer patients (2008–2018)

Authors [References]	Year	Main focus	Description/analysis	No. of databases	No. of included studies	Performed QA	Risk of bias assessment	Result(s)
Ballinger and Fallowfield [80]	2009	Assessment of QOL in older patients	Review	NA	25	No	No	The long-term survivorship studies indicate that older patients are perhaps better equipped mentally to deal with treatments
Munoz [81]	2010	Quality of life during treatment in young patients	Review	NA	NA	No	No	Patients who undergone mastectomy have worse body image and disturbances in their sexual life. Patients treated with mastectomy and adjuvant chemotherapy are those with the greatest impairment. Also, sexual activity is negatively affected by chemotherapy
Rosenberg and Partridge [82]	2013	QOL related to physical and psychosocial functioning in young premature menopause patients	Review	NA	NA	No	No	Effective strategies as an intervention should be applied to relieve symptoms and improve QOL in younger age groups
Samuel et al. [83]	2016	HRQOL in young black survivors	Systematic review	5	6	Yes	No	Young black BCS generally experience suboptimal HRQOL after breast cancer diagnosis

*QA* quality appraisal, *NA* not available, *HRQO* health-related quality of life, *QOL* quality of life, *BCS* breast cancer survivors

#### Assessment of quality of life among nations/races

A number of reviews [84–91] addressed the quality of life among breast cancer patients of different races for instance African American patients [84, 88], or among different nations such as Spanish breast cancer patients [85], Latina and non-Latina breast cancer survivors [86], Indian breast cancer patients [87], Arab women [89], Asian breast cancer patients [90] and Iranian breast cancer patients [91] (Table 6). Good scores of global health were recorded for in both African American and white survivors [84, 88], but it was reported that Latina breast cancer survivors on average experience worse QOL than non-Latina whites [86]. Asian breast cancer patients with comorbidities and those who treated with chemotherapy, having less social support, and with more unmet needs, had poorer HRQOL [90]. Limited information on quality of life in Arab women with breast cancer patients exist [89].

**Table 6:** A list of reviews on quality of life in different nations/races (2008–2018)

Authors [References]	Year	Main focus	Description/analysis	No. of databases	No. of included studies	Performed QA	Risk of bias assessment	Result(s)
Russell et al. [84]	2008	QOL of African American and white survivors	Review	4	26 qualitative and quantitative	No	No	QOL was different in two groups. Overall global quality of life was favorable in both African American and white survivors
Delgado-Sanz et al. [85]	2011	QOL in Spanish BC patients	Systematic review	8	25	Yes	No	Research into health-related quality of life of breast-cancer patients is a little developed
Yanez et al. [86]	2011	QOL among Latina breast cancer patients	Systematic review	2	22 qualitative and quantitative	No	No	Latina BCS on average experience worse QOL than non-Latina Whites
Deshpande et al. [87]	2013	QOL outcomes in Indian	Review	NA	NA	No	No	Clinical pharmacists may give the major support to Indian healthcare system in future
Mollica et al. [88]	2015	QOL in African American breast cancer survivors	Integrative literature review	5	19	Yes	No	Researchers must focus on factors from a multi-domain perspective to truly understand the varied dimensions influencing QOL
Haddou Rahou et al. [89]	2016	QOL in Arab women	Systematic review	5	13 qualitative and quantitative studies	Yes	No	Good scores of global health were recorded at Arab women compared to other countries. There was a difference in QOL scores and its associated factors among Arab women from different nations
Ho et al. [90]	2018	QOL in Asian patients	Systematic review	3	57	Yes	No	Patients with comorbidities and those treated with chemotherapy, with less social support and with more unmet needs, have poorer HRQOL
Bouya et al. [91]	2018	QOL of Iranian patients	Systematic review and Meta-analysis	4	18	Yes	No	Moderate level of QOL in patients was indicated

*QA* quality appraisal, *NA* not available, *QOL* quality of life, *BCS* breast cancer survivors

#### Qualitative reviews

Although some reviews included both quantitative and qualitative studies [84, 86, 89], there was only one review that exclusively reviewed qualitative studies [32]. The review included seven qualitative studies focusing on quality of life of breast cancer patients during and up to 10 years after treatment. Studies were from both developed and developing countries. The review generated three synthesized findings: to achieve effective care, clinicians are required to be aware of the impact of the disease and its treatment on physical and psychosocial domains of quality of life, for effective patient-centered care, they need to know about these effects on social relationships; finally, clinicians should be aware that women use religion and spirituality in order to cope with breast cancer treatment and to improve their own quality of life [32].

### Achievements so far and a brief synthesis

During 2008 to 2018 the number of reviews increased to 82 compared to 29 reviews during 1974 to 2007. This in fact is a reflection of the increase in the number of studies on quality of life among breast cancer patients worldwide. Of these, reviews emerging from less developed countries were evident. Even though the quality of these reviews was relatively poor, data from such studies surely provided more understanding on quality of life in breast cancer patients with different cultural backgrounds. According to the AMSTAR on average above 85% of publications had moderate to high quality, as we shown in Tables 1, 2, 3, 4, 5 and 6, but a considerable number of published reviews lacked standards for reporting, 56 out of 82 (68%) did not followed the PRISMA, 51% did not performed quality assessment, and 75% did not reported how risk of bias was evaluated. However, it is difficult to synthesis the data, we provided a tabulated summary of factors that might improve or decrease (worsen) quality of life in breast cancer patients. The summary is derived from review papers that included in this overview (Table 7).

**Table 7:** Factors related to improved or reduced quality of life in breast cancer patients and survivors

Factors that might improve quality of life	Factors that might deteriorate quality of life
Reduction of radiation-induced skin toxicity using simultaneous integrated boost, accelerated partial breast irradiation, and prone positioning	Adjuvant endocrine therapy-related side effects including vasomotor symptoms such as hot flashes
Adjuvant chemotherapy and radiotherapy in the elderly with solid tumors	Targeted therapy-related side effects: diarrhoea and skin rash in the adjuvant and metastatic settings for HER2+ breast cancer
Oncoplastic breast surgery	Body image after mastectomy
Both immediate and delayed breast reconstruction in long-term	Chemotherapy-induced alopecia
Better preoperative counseling and informed decision-making	Disturbances in sexual life
Physical activity interventions such as yoga, exercise, physical self-management, complementary exercise, art therapies, and mind–body exercise therapy	Less social support and unmet needs
Treatment of lymphedema: manual lymphatic drainage	Lymphedema affecting woman’s physical, psychological, and emotional well-being
Psychoeducational support or receiving social support in early stage breast cancer	Premature menopause, menopausal symptoms, and infertility
Cognitive behavioral therapy	Comorbid depression which significantly increases the burden of distress and dysfunction

## Discussion

### Patient reported outcomes

Instruments introduced to quantify quality of life in breast cancer patients have developed frequently over the last decade. From the health professionals’ and patients’ views among specific measures, the EORTC QLQ-BR23 and the FACIT-B were the most acceptable instruments. However, despite of reporting the good performance for these measures [17–20], others found that current instruments do not address important specific issues such as surgery‐specific conditions [10]. In addition, a recent review suggested that developing well-designed and more specific tools are needed to evaluate the side effects of novel therapies [21]. We believe that there is no need to develop new instruments, and rather if needed could add extra items to the existing questionnaires to fill the gaps as the EORTC quality of life study group did. They currently updated the EORTC QLQ-BR23 and introduced the QLQ-BR45 to cover all existing gaps. Two main reasons for this revision was indicated: the obvious changes in standard therapy and consequently emergence of new therapies that led to new different side effects, and the impacts of new drugs on patient’s quality of life [92, 93]. Above all we think the new direction for setting international standards for the analysis of quality of life and patient-reported outcomes in cancer trials is a step forward to prevent disorganized reporting, and to encourage using appropriate instruments to measure quality of life in cancer patients in general and in breast cancer patients in particular [94–96].

### Methodological issues

A number of reviews indicated that although methodological issues improved greatly, still reviews suffer from poor transparency in reporting on quality appraisal and risk of bias assessment. A review indicated that the sound scientific methodology in HRQOL was undermined by poorly designed and underpowered studies [11]. The current overview indicated that although all reviews have considered the principle components of AMSTAR checklist, the vast majority of reviews not included publication bias. However, the quality of reviews published during the last decade seems did not changed so much and thus that there is a need to further increase their quality. One way to achieve this might be registration of reviews in PROSPERO (International prospective register of systematic reviews).

### Treatment modalities

Quality of life can be an important predictor of better treatment outcomes [45]. A review, as an example, indicated that most studies reported increase in long-term satisfaction among patients who underwent surgery [48]. However, as a recent review suggests quality of life in breast cancer patients who receive surgery even might depend on decision-making process and communication style of care physicians. As such the review found that patients who received physician-dominated communication had lower physical function compared with those who took a more active role in their treatment choices processing [97]. This therefore sustains the need to increase the patients' information in order to prevent decisional regret, a common phenomenon after breast reconstruction [98]. In fact, this reflects a previous recommendation to clinicians that: there is a long life after breast cancer and clinicians should consider this while discussing treatment options with patients [33].

### Physical activity

Studies and reviews on physical activities have received much attention over the last decade. Reviews showed that interventions based on physical activities have not only improved breast cancer patients’ quality of life, but also could reduce symptoms including breast, arm and early menopausal symptoms [55, 75]. Moreover, positive effects and significant benefits of supervised combined aerobic resistance exercise on fatigue and QOL were reported in patients during their adjuvant radiotherapy [63]. Overall, one might argue that simple and inexpensive interventions or scheduling social events or even providing the green environments and neighborhoods might help to improve quality of life in breast cancer patients.

### Alternative therapies

Studies suggest that complementary and alternative therapies have achieved the potential of integrating into clinical practice [56]. However, according to the existing evidence with regard to CAM, yet, further high quality randomized clinical trials or longitudinal studies are required to evaluate net benefits of such treatments on quality of life of breast cancer patients [56, 63]. Yoga as a complementary therapy was frequently recommended in reviews. It seems that since practicing yoga as mind–body exercise could enhance psychological and social well-being, thus it could improve quality of life among breast cancer survivors [68]. Based on the quality of the evidence, for instance, an evidence (with moderate quality) supported the recommendation of yoga as a supportive intervention for improving HRQOL and reducing fatigue and sleep disturbances when compared with no therapy, as well as for reducing depression, anxiety and fatigue, when compared with psychosocial/educational interventions [62]. In spite of suggesting yoga in most studies, a review found that the most advantageous aspect of yoga is still not clear [57].

### Symptoms

Symptoms including anxiety, pain, fatigue and menopausal symptoms can significantly impact patients' daily live activities and subsequently their quality of life. It appears that the more affecting symptoms in breast cancer patients are still neglected topics in reviews. Studies are required to be carried out on symptoms’ burden and functional decline in breast cancer patients and survivors. The most frequently reported bothersome symptoms in breast cancer survivors were fatigue, insomnia, depression, cognitive dysfunction, reproductive and menopausal symptoms, and lymphedema [33]. Physical, psychological and emotional well-being of breast cancer patients are affected by lymphedema [53]. Reviews referenced to the treatment of lymphedema indicated that depending on the type of therapy such as manually lymphatic drainage or combined decongestive therapy, a significant positive impact on patient’s quality of life is observed [16, 53], although recently it has been suggested that still there is a need for high-quality evidence to talk about the effectiveness of combined decongestive therapy [54].

### Psychological interventions

‘The day you lose your hope is the day you start to die’ is a key sentence that implies the key role for psychological interventions in improving breast cancer symptoms and enhancing patients’ quality of life [99]. Psycho-educational support, for example, in improving breast cancer symptoms and in improving emotional well-being is an effective intervention [76]. Moderate to strong evidence reported a relationship between fatigue and depression, anxiety, pain, sleep disturbances, insufficient physical activity, and difficulties with coping with cancer, all of which can be addressed in psychological interventions [79]. Cognitive behavior therapy as an effective therapy in reducing symptoms and in improving QOL and psychological health of survivors has been reported [78]. Interestingly, it can be seen that joint effect of behavioral techniques and physical exercise can improve psychosocial functioning and HRQOL in breast cancer patients and survivors further [74]. In addition, as recently suggested, specific mindfulness activities also might help patients of all ages to cope with their diagnosis [99].

### The elderly and quality of life

Overall, we found that elderly patients reported moderate to good quality of life. Older patients are perhaps better equipped mentally to deal with treatments compared to younger patients [80]. According to the findings of a review, the impact of local or systemic treatments on QOL in the elderly early-breast cancer patients was maintained or improved [31] or adjuvant chemotherapy and radiotherapy did not have detrimental effects on QOL in most elderly patients with solid tumors [52].

## Limitations and the future directions

One should note that this review of reviews has some limitations. The main critic is the fact that it is difficult to evaluate in what way the results add to existing knowledge since 82 reviews with different objectives were evaluated. While a more focused and in-depth reviews are recommended, it is useful to bear in mind that this review of reviews in fact is a bibliometric analysis of review papers and provides a representation of what achieved during the last decade studying quality of life in breast cancer patients. We believe this even could highlight repetitions, discrepancies, and indicate areas that require more investments. For instance, we noticed that although reviews included both breast cancer patients and survivors, no specific reviews on quality of life in breast cancer survivors could be identified. Perhaps this is an area that could be addressed independently since there are differences in quality of life between newly diagnosed breast cancer patients, patients who are receiving different treatments, and the long-term survivors who successfully completed their treatments and now they have back to normal life. Survivorship in breast cancer patients is a very important and relevant topic and deserves more attention. Finally, it is important to notice that this review of reviews did not separate the interventional studies from other types of studies (usually descriptive or correlational). Perhaps a better organization might be to reporting reviews based on separate objectives. However, we have tried to provide a summary table (Table 7) which could help to identify factors that might improve or deteriorate quality of life in breast cancer patients.

## Conclusion

Quality of life in breast cancer patients improved greatly during recent years as several simple but effective interventions such as physical activity and psychosocial interventions proved to be effective. However, symptoms caused by different treatment modalities are still under estimation and need more serious attention. Pain, lymphedema, worry, sexual function especially for young patients, and the future outlooks all are among issues that deserve further consideration in order to improve quality of life in breast cancer patients.

## Supplementary information

**Additional file 1.** List of reviews from 1974–2007.

## Data Availability

Not applicable.

## Abbreviations

CDSR: Cochrane Database of Systematic Reviews
PRISMA: Preferred reporting items for systematic reviews and meta-analyses
AMSTAR: A Measurement Tool to Assess systematic Reviews
PROSPERO: International prospective register of systematic reviews
QOL: Quality of life
HRQOL: Health related quality of life
CAM: Complementary and alternative medicine
