# Correction to: Health-related quality of life in breast cancer patients: review of reviews from 2008 to 2018

**DOI:** 10.1186/s12955-022-01942-w

**Published:** 2022-02-25

**Authors:** Parisa Mokhtari-Hessari, Ali Montazeri

**Affiliations:** 1 Integrative Oncology Research Group, Breast Cancer Research Center, Motamed Cancer Institute, ACECR, Tehran, Iran; 2 Population Health Research Group, Health Metrics Research Center, Iranian Institute for Health Sciences Research, ACECR, Tehran, Iran; 3 Faculty of Humanity Sciences, University of Science and Culture, ACECR, Tehran, Iran

## Correction to: Health Qual Life Outcomes (2020) 18:338 10.1186/s12955-020-01591-x

The original article contained an error in co-author, Parisa Mokhtari-Hessari’s name which has since been corrected.

