# Supplementary material for: Health-related quality of life in breast cancer patients: review of reviews from 2008 to 2018
Source: Health Qual Life Outcomes. 2020 Oct 12;18:338. doi: 10.1186/s12955-020-01591-x (PMC7552560; doi:10.1186/s12955-020-01591-x)
Supplement: Supplementary file 1 — Additional file 1. List of reviews from 1974–2007. [file 12955_2020_1591_MOESM1_ESM.doc]

**1:** A list of some overview papers on quality of life in breast cancer patients (1974-2007)

| **Author(s)** | **Year** | **Main focus** | **Conclusion(s)** |
| --- | --- | --- | --- |
| McEvoy and McCorkle | 1990 | QOL in advanced breast cancer | Efforts to manage advanced breast cancer must include both current medical therapies and attention to the critical factors associated with enhancing their QOL. |
| Kiebert et al. | 1991 | Impact of breast conserving surgery vs. mastectomy on QOL | There were no substantial differences between the two treatment modalities except for body image and sexual functioning in favor of breast conserving surgery. |
| Aarenson | 1993 | Assessments of QOL and benefits from adjuvant therapies | Adjuvant therapies could improve QOL in breast cancer patients. |
| Bryson and Plosker | 1993 | Tamoxifen as adjuvant therapy | Tamoxifen has a low cost-utility ratio in postmenopausal women with node-positive, estrogen receptor-positive breast cancer. |
| Stefanek | 1994 | QOL research, provider-patient communication, and psychological distress of spouses and other relatives of breast cancer patients | This review summarizes and critiques publications in three identified areas. |
| Ganz | 1994 | Review of various approaches to the measurement of QOL, the important QOL issues in the treatment of breast cancer, and what is known about QOL of older women with breast cancer | Ongoing and future research using newer approaches to QOL assessment should provide additional information on this important topic. |
| Osoba | 1994 | QOL as a treatment endpoint | Advances in understanding HRQOL in metastatic breast cancer will aid the development of rational treatment policies. |
| Carlson | 1998 | QOL in metastatic breast cancer | Clinician must balance anti-tumor activity, performance status, and the usual toxicity measures as surrogates for QOL associated with each specific therapy. |
| Leedham and Ganz | 1999 | Psychological concerns and mental health | Psychological concerns and mental health are important issues for breast cancer patients and should be recognized and treated when necessary. |
| Rustoen and Begnum | 2000 | Nursing practice | Nurses play an important role in meeting the needs of breast cancer patients. |
| Shapiro et al. | 2001 | Relationship between psychosocial variables and QOL | A broader, more integrative framework that includes psychosocial factors is needed to evaluate breast cancer consequences. |
| Partridge et al. | 2001 | QOL before, during and after high-dose chemotherapy | Resulting transient impaired overall QOL with subsequent improvement over time. |
| Kurtz and Dufour | 2002 | QOL in older patients with metastatic disease receiving either standard treatment or new drugs | Aromatase inhibitors (such as taxanes and orally administered chemotherapy) provide similar or a better QOL as compared to first line endocrine therapy with tamoxifen. |
| Costantino | 2002 | Hormonal treatments in metastatic breast cancer patients | QOL data is useful for both clinicians and patients in evaluating treatment options and developing treatment strategies. |
| Fallowfield | 2004 | Hormonal therapies | Tolerability profiles of available treatment options are highlighted. |
| Sammarco | 2004 | QOL of older breast cancer patients | Outpatient and long-term care should become a key setting for implementation of QOL interventions for women with breast cancer. |
| Knobf | 2006 | Endocrine effects of adjuvant therapy in younger survivors | Causes premature menopause that is associated with poorer QOL, decreased sexual functioning, menopausal symptom distress, psychosocial distress related to infertility, and infertility. |
| Kayl and Meyers | 2006 | Side effects of chemotherapy | QOL issues may help to guide patient-care decision. |
| Diel | 2007 | Effectiveness of bisphosphonates on bone pain and quality of life in breast cancer patients with metastatic bone disease | Clinical trial data demonstrate that bisphosphonates offer significant and sustained relief from bone pain and can also improve quality of life in patients with metastatic breast cancer. New treatment schedules using high dose bisphosphonates can offer rapid relief of acute, and severe bone pain. |
| Rozenberg et al. | 2007 | Co-morbid conditions and breast cancer | Women with breast cancer and three or more co-morbid conditions have a 20-fold higher rate of mortality from causes other than breast cancer and a 4-fold higher rate of all-cause mortality when compared with patients who have none. |

**2:** A list of systematic reviews on different aspects of quality of life in breast cancer patients (1974-2006)

| **Author(s)** | **Year** | **Main focus** | **Conclusion(s)** |
| --- | --- | --- | --- |
| Irwig and Bennetts | 1997 | A systematic review of quality of life after breast conservation or mastectomy | Apart body image it is unclear whether breast conservation or mastectomy results in better psychosocial outcomes. |
| Bottomley and Therasse | 2002 | Systemic therapy (chemotherapy, hormonal therapy, or biological therapy) in advanced breast cancer (1995-2001) | QOL data provide invaluable insights into the treatment and care of patients. |
| Shimozuma et al. | 2002 | Systematic overview of the literature (1982-1999) | To date there have been almost no appropriate systematic overviews or guidelines issued for QOL assessment studies related to breast cancer. |
| Goodwin et al. | 2003 | Randomized clinical trials of treatment (review of literature from 1980-2001) | Until results of ongoing trials in breast cancer are available, caution is recommended in initiating new QOL studies unless treatment equivalency is expected or unless unique or specific issues can be addressed. |
| Rietman et al. | 2003 | Late morbidity of breast cancer (review of literature from 1980 to 2000) | Significant relationship between late morbidity and restrictions of daily activities and poorer QOL was reported. |
| Payne et al. | 2003 | Racial disparities in the palliative care for African-American (review of literature from 1985 to 2000) | Differences in treatment patterns, pain management, and hospice care exist between African-American and other ethnic groups. |
| Fossati | 2004 | Randomized clinical trials of cytotoxic or hormonal treatments in advanced breast cancer (review of published literature before Dec 2003 | QOL assessments added relatively little value to classical clinical endpoints. |
| Mols et al. | 2005 | Systematic review among long-term survivors | Focusing on the long-term effects of breast cancer is important when evaluating the full extent of cancer treatment. |
| Grimison and Stockler | 2007 | Adjuvant systemic therapy for early-stage breast cancer (review of literature from 1996 to Feb. 2007) | For the majority of breast cancer patients most aspects of health-related quality of life recover after adjuvant chemotherapy ends without long-term effects except vasomotor symptoms and sexual dysfunction. |
